# Crystal structure of 4-meth­oxy­phenyl 2-oxo-2*H*-chromene-3-carboxyl­ate

**DOI:** 10.1107/S2056989015006970

**Published:** 2015-05-07

**Authors:** H.C. Devarajegowda, P. A. Suchetan, S. Sreenivasa, H. T. Srinivasa, B. S. Palakshamurthy

**Affiliations:** a Department of Physics, Yuvaraja’s College (Constituent College), University of Mysore, Mysore, Karnataka 570 005, India; b Department of Studies and Research in Chemistry, U.C.S., Tumkur University, Tumkur, Karnataka 572 103, India; c Raman Research Institute, C. V. Raman Avenue, Sadashivanagar, Bangalore, Karnataka, India

**Keywords:** crystal structure, 2-oxo-2*H*-chromene, C—H⋯π inter­actions, C—H⋯O inter­actions

## Abstract

In the title compound, C_17_H_12_O_5_, the dihedral angle between the planes of the coumarin ring system (r.m.s. deviation = 0.015 Å) and the benzene ring is 48.04 (10)°. The central CO_2_ group subtends a dihedral angle of 27.15 (11)° with the coumarin ring system and 74.86 (13)° with the benzene ring. In the crystal, mol­ecules are linked by C—H⋯O inter­actions, which generate a three-dimensional network. Very weak C—H⋯π inter­actions are also observed.

## Related literature   

For details of the biological activies of 2-oxo-2*H*-chromene derivatives, see: Kawase *et al.* (2001 ▸); Traven (2004 ▸); Lacy & O’Kennedy (2004 ▸); Chimenti *et al.* (2009 ▸). For related structures, see: Sreenivasa *et al.* (2013 ▸); Devarajegowda *et al.*, (2013 ▸).
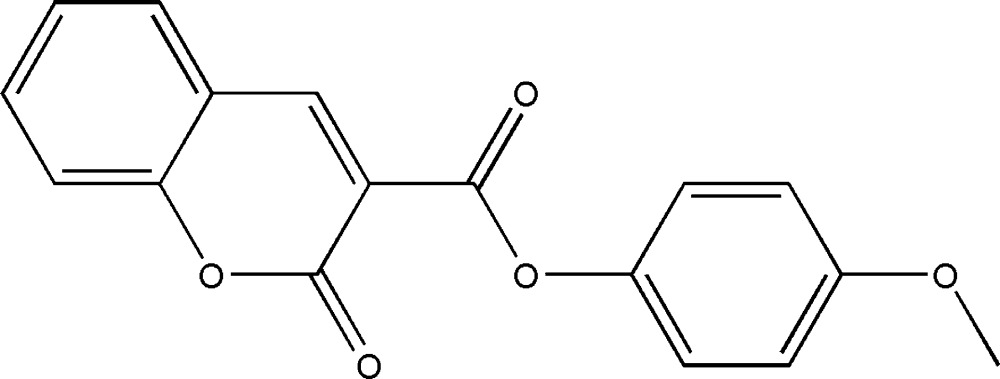


## Experimental   

### Crystal data  

C_17_H_12_O_5_
*M* *_r_* = 296.27
Orthorhombic, 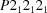

*a* = 6.2648 (18) Å
*b* = 10.435 (3) Å
*c* = 20.621 (7) Å
*V* = 1348.0 (7) Å^3^
*Z* = 4
Mo *K*α radiation
μ = 0.11 mm^−1^
*T* = 296 K
0.22 × 0.20 × 0.18 mm

### Data collection  

Bruker APEXII CCD diffractometer
Absorption correction: multi-scan (*SADABS*; Bruker, 2013 ▸) *T*_min_ = 0.977, *T*_max_ = 0.981
10472 measured reflections
2385 independent reflections
2150 reflections with *I* > 2σ(*I*)
*R*_int_ = 0.050

### Refinement  

*R*[*F* ^2^ > 2σ(*F* ^2^)] = 0.035
*wR*(*F* ^2^) = 0.085
*S* = 1.09
1411 reflections
201 parameters
H-atom parameters constrained
Δρ_max_ = 0.16 e Å^−3^
Δρ_min_ = −0.16 e Å^−3^

### 

Data collection: *APEX2* (Bruker, 2013 ▸); cell refinement: *SAINT* (Bruker, 2013 ▸); data reduction: *SAINT* (Bruker, 2013 ▸); program(s) used to solve structure: *SHELXS97* (Sheldrick, 2008 ▸); program(s) used to refine structure: *SHELXL2014* (Sheldrick, 2015 ▸); molecular graphics: *Mercury* (Macrae *et al.*, 2008 ▸); software used to prepare material for publication: *SHELXL2014* (Sheldrick, 2015 ▸).

## Supplementary Material

Crystal structure: contains datablock(s) I, New_Global_Publ_Block. DOI: 10.1107/S2056989015006970/hb7399sup1.cif

Structure factors: contains datablock(s) I. DOI: 10.1107/S2056989015006970/hb7399Isup2.hkl

Supporting information file. DOI: 10.1107/S2056989015006970/hb7399Isup3.cml

**Fig. 1.**
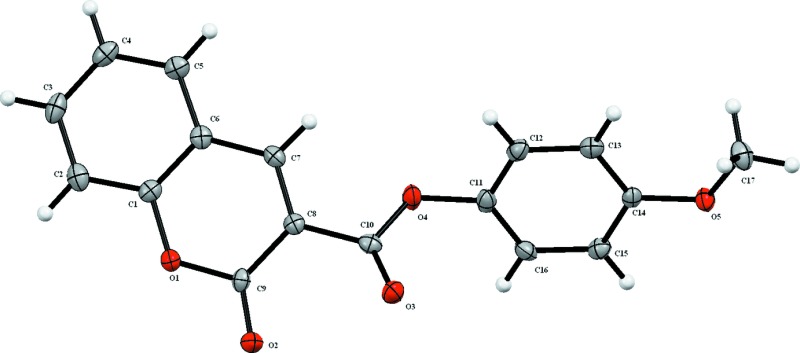
The molecular structure of the title compound, showing displacement ellipsoids drawn at the 50% probability level.

**Fig. 2.**
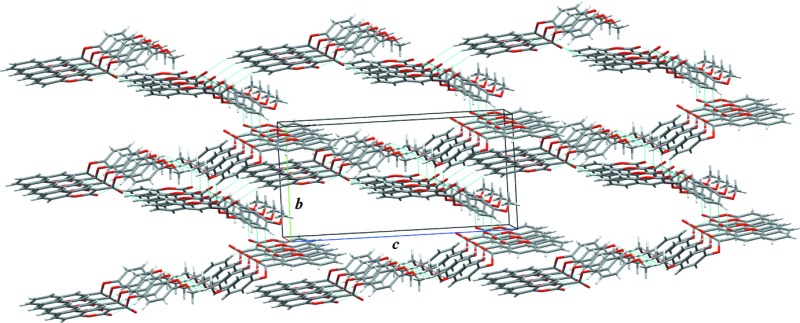
The packing of (I) showing grid like structure when viewed along a axis.

**Fig. 3.**
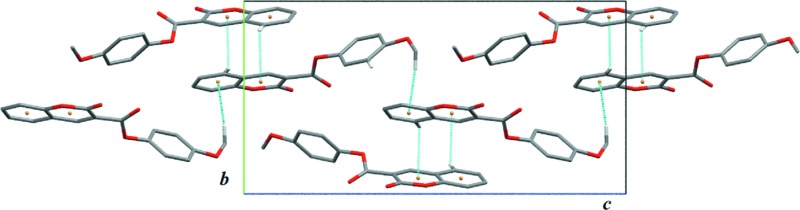
The packing of (I) showing C—H···π interactions when viewed along a axis.

. DOI: 10.1107/S2056989015006970/hb7399fig1.tif
The mol­ecular structure of the title compound, showing displacement ellipsoids drawn at the 50% probability level.

a . DOI: 10.1107/S2056989015006970/hb7399fig2.tif
The packing of (I) showing grid like structure when viewed along *a* axis.

a . DOI: 10.1107/S2056989015006970/hb7399fig3.tif
The packing of (I) showing C—H⋯π inter­actions when viewed along *a* axis.

**Fig. 4.**
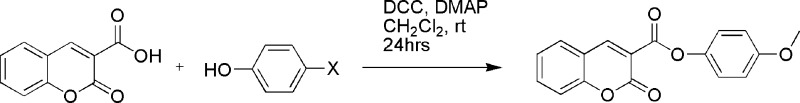
The formation of the title compound.

. DOI: 10.1107/S2056989015006970/hb7399fig4.tif
The formation of the title compound.

CCDC reference: 1058259

Additional supporting information:  crystallographic information; 3D view; checkCIF report

## Figures and Tables

**Table 1:** Hydrogen-bond geometry (, ) *Cg*1 and *Cg*2 are the centroids of the C1/C6/C7/C8/C9/O1 and C1/C2/C3/C4/C5/C6 rings, respectively.

*D*H*A*	*D*H	H*A*	*D* *A*	*D*H*A*
C17H17*B*O5^i^	0.96	2.50	3.228(3)	132
C12H12O2^ii^	0.93	2.48	3.353(3)	156
C15H15O2^iii^	0.93	2.50	3.207(3)	133
C3H3O3^iv^	0.93	2.47	3.272(4)	145
C5H5*Cg*1^v^	0.93	2.82	3.303 (3)	114
C17H17*C* *Cg*2^vi^	0.93	2.96	3.709 (4)	136

Symmetry codes: (i) 
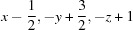
; (ii) 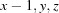; (iii) 
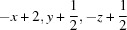
; (iv) 
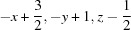
; (v) 
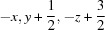
; (vi) 
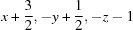
.

## supplementary crystallographic information

### S1. Chemical context

The 2-oxo-2H-chromene is a useful starting material for the construction of heterocyclic compounds with a broad spectrum of biological activities. Especially the 3-substituted derivatives exhibits pharmacological effects such as analgesic, anti-arthritis, anti-inflammatory, anti-pyretic, anti-viral, anti-cancer and anti­coagulant properties (Chimenti *et al.*, 2009; Traven *et al.*, 2004; Lacy *et al.*, 2004). Moreover, these derivatives are well known for their anti-microbial activity toward different microorganisms, they show anti-microbial activity with reference to anti-H. *pylori activity*.(Kawase *et al.*, 2001).

2-oxo-2H-chromenes (coumarins) have been also used in the field of medicine, cosmetics and fluorescent dyes. They are efficient fluoro­phores characterized by good emission quantum yields and are used as materials for lasers in organic light emitting devices, non-linear optical chromophores and fluorescent labels. Keeping these facts in mind and in continuation of our work on 2-oxo-2H-chromene derivatives (Sreenivasa *et al.*, 2013; Palakshamurthy, Sreenivasa *et al.*, 2013; Palakshamurthy, Devarajegowda *et al.*, 2013; Devarajegowda, *et al.*,2013), herein we report the synthesis and crystal structure of 4-Meth­oxy­phenyl 2-oxo-2H-chromene-3-carboxyl­ate (I).

### S2. Structural commentary

In the title molecule (I), C_17_H_12_O_5_, the coumarin ring is almost planar, the rms deviation (considering non Hydrogen atom) being 0.012 (1)Å. The dihedral angle between the coumarin ring and the phenyl ring in (I) is 48.04 (10)^o^. Compared to this, the dihedral angle is 21.11 (1)° in 4-(octyl­oxy)phenyl 2-oxo-2H-chromene-3 -carboxyl­ate (II) (Palakshamurthy, Devarajegowda *et al.*, 2013), 62.97 (2)^o^ in 4-(decyl­oxy)phenyl 7-(tri­fluoro­methyl)-2-oxo-2H-chromene-3-carboxyl­ate (III) (Palakshamurthy, Sreenivasa *et al.*, 2013b), 22.95 (11)^o^ in 4'-Cyano­biphenyl-4-yl 7-di­ethyl­amino- 2-oxo-2H-chromene-3-carboxyl­ate (IV) (Sreenivasa *et al.*, 2013) and 54.46 (17)^o^ in 4-[4-(Heptyl­oxy)benzoyl­oxy] phenyl 2-oxo-7- tri­fluoro­methyl-2H-chromene-3- carboxyl­ate (V) (Devarajegowda, *et al.*, 2013). Further, in (I), the dihedral angle between the central ester chain [C8—C10(O3)—O4] and the phenyl ring and the coumarin ring are 74.86 (10)^o^ and 27.16 (8)^o^ respectively. The meth­oxy group is slightly out of plane from the attached benzene ring, the C17—O5—C14—C13 torsion being 10.3 (3)^o^.

### S3. Supra­molecular features

In the crystal structure, the molecules are linked into zig-zag C9 chains along *c* axis via C3—H3···O3 inter­molecular inter­actions. Further, C12—H12···O2 inter­actions between the molecules in the neighbouring chains leads to C8 chains along *a* axis, and thus forming sheets in the *ac* plane. These sheets are inter­connected via an inter­molecular C15—H15···O2 inter­actions which form helical C7 chains running along *b* axis, and hence a three dimensional architecture is displayed. An additional C17—H17B···O5 inter­actions between the molecules in the neighbouring C7 helical chains leading to the formation of C3 chains along *a* axis results in sheets along *ab* plane. Thus, a grid like three dimensional structure is observed. Packing of the molecules displaying the columns formed along *a* axis is shown in Figure 2.

The packing also features C5—H5···Cg1 and C17—H17C···Cg2 inter­actions (where Cg1 and the Cg2 are the centroids of the rings C1/C6/C7/C8/C9/O1 and C1/C2/C3/C4/C5/C6 respectively), as shown in Figure 3.

### S4. Synthesis and crystallization

A solution of di­cyclo­hexyl­carbodi­imide (DCC) dissolved in dried CH_2_Cl_2_ was added to a solution containing coumarin 3-carb­oxy­lic acid (1.0 mmol) and 4-meth­oxy­phenol (1.0 mmol) and a catalytic amount of N—N-Di­methyl­amino­pyrimidine (DMAP) in anhydrous di­chloro­methane (CH_2_Cl_2_), under stirring, After 24 hrs of stirring, di­cyclo­hexyl­urea was filtered off and the solution was concentrated. The solid residue was purified by column chromatography on silica gel (60–120) using chloro­form (CHCl_3_) as an eluent. Colourless prisms of the title compound were grown by slow evaporation of an ethanol solution at room temperature.

### S5. Refinement details

Crystal data, data collection and structure refinement details are summarized in Table 1. The H atoms were positioned with idealized geometry using a riding model with C—H = 0.93-0.99 Å. All H-atoms were refined with isotropic displacement parameters (set to 1.2-1.5 times of the U eq of the parent atom).

### Crystal data

**Table:**

C_17_H_12_O_5_	*D*_x_ = 1.460 Mg m^−^^3^
*M**_r_* = 296.27	Melting point: 435 K
Orthorhombic, *P*2_1_2_1_2_1_	Mo *K*α radiation, λ = 0.71073 Å
*a* = 6.2648 (18) Å	Cell parameters from 2385 reflections
*b* = 10.435 (3) Å	θ = 2.0–25.0°
*c* = 20.621 (7) Å	µ = 0.11 mm^−^^1^
*V* = 1348.0 (7) Å^3^	*T* = 296 K
*Z* = 4	Prism, colourless
*F*(000) = 616	0.22 × 0.20 × 0.18 mm
prism	

### Data collection

**Table:**

Bruker APEXII CCD diffractometer	2385 independent reflections
Radiation source: fine-focus sealed tube	2150 reflections with *I* > 2σ(*I*)
Graphite monochromator	*R*_int_ = 0.050
Detector resolution: 2.01 pixels mm^-1^	θ_max_ = 25.0°, θ_min_ = 2.0°
phi and ω scans	*h* = −7→5
Absorption correction: multi-scan (*SADABS*; Bruker, 2013)	*k* = −12→12
*T*_min_ = 0.977, *T*_max_ = 0.981	*l* = −24→23
10472 measured reflections	

### Refinement

**Table:**

Refinement on *F*^2^	Secondary atom site location: difference Fourier map
Least-squares matrix: full	Hydrogen site location: inferred from neighbouring sites
*R*[*F*^2^ > 2σ(*F*^2^)] = 0.035	H-atom parameters constrained
*wR*(*F*^2^) = 0.085	*w* = 1/[σ^2^(*F*_o_^2^) + (0.0342*P*)^2^ + 0.2933*P*] where *P* = (*F*_o_^2^ + 2*F*_c_^2^)/3
*S* = 1.09	(Δ/σ)_max_ < 0.001
1411 reflections	Δρ_max_ = 0.16 e Å^−^^3^
201 parameters	Δρ_min_ = −0.16 e Å^−^^3^
0 restraints	Extinction correction: *SHELXL2014* (Sheldrick, 2015), Fc^*^=kFc[1+0.001xFc^2^λ^3^/sin(2θ)]^-1/4^
0 constraints	Extinction coefficient: 0.010 (3)
Primary atom site location: structure-invariant direct methods	

### Special details

**Table:**

Geometry. All e.s.d.'s (except the e.s.d. in the dihedral angle between two l.s. planes) are estimated using the full covariance matrix. The cell e.s.d.'s are taken into account individually in the estimation of e.s.d.'s in distances, angles and torsion angles; correlations between e.s.d.'s in cell parameters are only used when they are defined by crystal symmetry. An approximate (isotropic) treatment of cell e.s.d.'s is used for estimating e.s.d.'s involving l.s. planes.

### Fractional atomic coordinates and isotropic or equivalent isotropic displacement parameters (Å^2^)

**Table:**

	*x*	*y*	*z*	*U*_iso_*/*U*_eq_	
O5	0.2218 (3)	0.80557 (17)	0.42819 (9)	0.0181 (5)	
O4	0.5756 (3)	0.70623 (17)	0.18810 (9)	0.0213 (5)	
O1	1.0215 (3)	0.54316 (17)	0.02385 (9)	0.0172 (5)	
O2	1.1382 (3)	0.53880 (19)	0.12431 (9)	0.0221 (5)	
O3	0.8203 (3)	0.56290 (18)	0.22324 (9)	0.0216 (5)	
C17	0.0344 (5)	0.7406 (3)	0.44957 (15)	0.0235 (7)	
H17A	−0.0827	0.7619	0.4215	0.035*	
H17B	0.0014	0.7664	0.4931	0.035*	
H17C	0.0584	0.6497	0.4484	0.035*	
C14	0.3033 (5)	0.7725 (2)	0.36853 (13)	0.0146 (6)	
C13	0.1987 (5)	0.6954 (2)	0.32378 (13)	0.0185 (6)	
H13	0.0656	0.6606	0.3333	0.022*	
C12	0.2960 (5)	0.6709 (2)	0.26457 (14)	0.0195 (7)	
H12	0.2284	0.6191	0.2341	0.023*	
C11	0.4914 (5)	0.7230 (2)	0.25097 (13)	0.0189 (6)	
C10	0.7332 (4)	0.6180 (2)	0.17961 (14)	0.0164 (6)	
C8	0.7751 (4)	0.6021 (2)	0.10923 (13)	0.0143 (6)	
C9	0.9873 (5)	0.5583 (2)	0.08977 (13)	0.0153 (6)	
C1	0.8638 (5)	0.5612 (2)	−0.02156 (13)	0.0155 (6)	
C2	0.9148 (5)	0.5377 (2)	−0.08562 (14)	0.0204 (7)	
H2	1.0497	0.5080	−0.0969	0.024*	
C3	0.7610 (5)	0.5592 (3)	−0.13272 (14)	0.0223 (7)	
H3	0.7935	0.5448	−0.1761	0.027*	
C7	0.6224 (5)	0.6229 (2)	0.06469 (13)	0.0148 (6)	
H7	0.4887	0.6511	0.0781	0.018*	
C6	0.6609 (4)	0.6025 (2)	−0.00282 (13)	0.0153 (6)	
C5	0.5082 (5)	0.6226 (2)	−0.05175 (13)	0.0181 (6)	
H5	0.3717	0.6500	−0.0407	0.022*	
C4	0.5591 (5)	0.6021 (3)	−0.11558 (14)	0.0204 (7)	
H4	0.4575	0.6169	−0.1476	0.024*	
C15	0.5015 (5)	0.8245 (2)	0.35439 (13)	0.0173 (6)	
H15	0.5705	0.8756	0.3849	0.021*	
C16	0.5972 (5)	0.8008 (3)	0.29511 (14)	0.0187 (7)	
H16	0.7294	0.8361	0.2851	0.022*	

### Atomic displacement parameters (Å^2^)

**Table:**

	*U*^11^	*U*^22^	*U*^33^	*U*^12^	*U*^13^	*U*^23^
O5	0.0178 (11)	0.0206 (9)	0.0160 (11)	−0.0012 (9)	0.0043 (8)	−0.0010 (8)
O4	0.0249 (12)	0.0249 (10)	0.0140 (11)	0.0105 (9)	0.0029 (8)	0.0000 (8)
O1	0.0158 (11)	0.0205 (9)	0.0154 (11)	0.0007 (9)	0.0018 (8)	−0.0017 (8)
O2	0.0175 (12)	0.0299 (11)	0.0189 (11)	0.0057 (9)	−0.0032 (9)	−0.0045 (8)
O3	0.0218 (12)	0.0276 (10)	0.0153 (11)	0.0042 (10)	−0.0001 (9)	0.0035 (8)
C17	0.0247 (19)	0.0205 (14)	0.0252 (17)	−0.0036 (13)	0.0078 (14)	0.0008 (12)
C14	0.0177 (16)	0.0133 (12)	0.0128 (15)	0.0039 (12)	0.0004 (12)	0.0022 (10)
C13	0.0173 (16)	0.0183 (13)	0.0198 (16)	0.0002 (13)	−0.0019 (13)	0.0006 (12)
C12	0.0230 (18)	0.0187 (14)	0.0169 (16)	0.0033 (13)	−0.0034 (13)	−0.0039 (11)
C11	0.0223 (17)	0.0198 (14)	0.0146 (15)	0.0070 (13)	0.0012 (12)	0.0007 (12)
C10	0.0139 (16)	0.0148 (13)	0.0204 (17)	−0.0021 (12)	0.0000 (13)	0.0012 (12)
C8	0.0154 (16)	0.0114 (12)	0.0163 (15)	−0.0017 (11)	0.0004 (12)	−0.0005 (11)
C9	0.0195 (17)	0.0125 (12)	0.0138 (15)	−0.0008 (12)	0.0019 (13)	−0.0013 (10)
C1	0.0186 (17)	0.0118 (12)	0.0162 (15)	−0.0028 (12)	−0.0013 (12)	0.0016 (11)
C2	0.0233 (17)	0.0151 (13)	0.0228 (17)	−0.0018 (12)	0.0056 (13)	−0.0018 (12)
C3	0.034 (2)	0.0186 (13)	0.0145 (16)	−0.0064 (13)	0.0031 (14)	−0.0009 (12)
C7	0.0135 (16)	0.0108 (12)	0.0202 (17)	−0.0005 (11)	0.0021 (12)	−0.0003 (11)
C6	0.0183 (17)	0.0097 (11)	0.0178 (16)	−0.0030 (12)	0.0017 (13)	0.0010 (11)
C5	0.0211 (17)	0.0127 (13)	0.0206 (16)	0.0006 (12)	−0.0020 (14)	0.0011 (11)
C4	0.0298 (19)	0.0143 (13)	0.0171 (16)	−0.0027 (13)	−0.0056 (13)	0.0017 (11)
C15	0.0188 (16)	0.0143 (12)	0.0189 (15)	−0.0001 (12)	−0.0030 (13)	−0.0019 (11)
C16	0.0125 (15)	0.0220 (14)	0.0216 (16)	0.0013 (12)	0.0020 (12)	0.0013 (12)

### Geometric parameters (Å, º)

**Table:**

O5—C14	1.376 (3)	C10—C8	1.484 (4)
O5—C17	1.426 (3)	C8—C7	1.344 (4)
O4—C10	1.361 (3)	C8—C9	1.462 (4)
O4—C11	1.411 (3)	C1—C2	1.381 (4)
O1—C1	1.374 (3)	C1—C6	1.397 (4)
O1—C9	1.385 (3)	C2—C3	1.386 (4)
O2—C9	1.201 (3)	C2—H2	0.9300
O3—C10	1.199 (3)	C3—C4	1.388 (4)
C17—H17A	0.9600	C3—H3	0.9300
C17—H17B	0.9600	C7—C6	1.429 (4)
C17—H17C	0.9600	C7—H7	0.9300
C14—C15	1.386 (4)	C6—C5	1.406 (4)
C14—C13	1.389 (4)	C5—C4	1.371 (4)
C13—C12	1.388 (4)	C5—H5	0.9300
C13—H13	0.9300	C4—H4	0.9300
C12—C11	1.368 (4)	C15—C16	1.384 (4)
C12—H12	0.9300	C15—H15	0.9300
C11—C16	1.388 (4)	C16—H16	0.9300
			
C14—O5—C17	117.6 (2)	O1—C9—C8	116.5 (2)
C10—O4—C11	118.2 (2)	O1—C1—C2	117.5 (3)
C1—O1—C9	122.8 (2)	O1—C1—C6	120.5 (2)
O5—C17—H17A	109.5	C2—C1—C6	122.0 (3)
O5—C17—H17B	109.5	C1—C2—C3	118.7 (3)
H17A—C17—H17B	109.5	C1—C2—H2	120.6
O5—C17—H17C	109.5	C3—C2—H2	120.6
H17A—C17—H17C	109.5	C2—C3—C4	120.5 (3)
H17B—C17—H17C	109.5	C2—C3—H3	119.8
O5—C14—C15	115.0 (2)	C4—C3—H3	119.8
O5—C14—C13	124.4 (3)	C8—C7—C6	121.4 (3)
C15—C14—C13	120.7 (3)	C8—C7—H7	119.3
C12—C13—C14	118.9 (3)	C6—C7—H7	119.3
C12—C13—H13	120.5	C1—C6—C5	117.8 (3)
C14—C13—H13	120.5	C1—C6—C7	118.0 (3)
C11—C12—C13	120.0 (3)	C5—C6—C7	124.2 (3)
C11—C12—H12	120.0	C4—C5—C6	120.5 (3)
C13—C12—H12	120.0	C4—C5—H5	119.8
C12—C11—C16	121.7 (3)	C6—C5—H5	119.8
C12—C11—O4	118.3 (3)	C5—C4—C3	120.5 (3)
C16—C11—O4	119.8 (3)	C5—C4—H4	119.8
O3—C10—O4	123.9 (3)	C3—C4—H4	119.8
O3—C10—C8	126.8 (3)	C16—C15—C14	120.2 (3)
O4—C10—C8	109.2 (2)	C16—C15—H15	119.9
C7—C8—C9	120.7 (3)	C14—C15—H15	119.9
C7—C8—C10	121.6 (3)	C15—C16—C11	118.5 (3)
C9—C8—C10	117.7 (2)	C15—C16—H16	120.7
O2—C9—O1	116.2 (3)	C11—C16—H16	120.7
O2—C9—C8	127.3 (3)		
			
C17—O5—C14—C15	−170.9 (2)	C9—O1—C1—C6	3.2 (3)
C17—O5—C14—C13	10.3 (4)	O1—C1—C2—C3	−177.9 (2)
O5—C14—C13—C12	178.8 (2)	C6—C1—C2—C3	1.8 (4)
C15—C14—C13—C12	0.1 (4)	C1—C2—C3—C4	−0.8 (4)
C14—C13—C12—C11	−0.3 (4)	C9—C8—C7—C6	0.3 (4)
C13—C12—C11—C16	0.0 (4)	C10—C8—C7—C6	−177.4 (2)
C13—C12—C11—O4	−174.0 (2)	O1—C1—C6—C5	178.3 (2)
C10—O4—C11—C12	−103.6 (3)	C2—C1—C6—C5	−1.4 (4)
C10—O4—C11—C16	82.4 (3)	O1—C1—C6—C7	−1.1 (3)
C11—O4—C10—O3	−8.0 (4)	C2—C1—C6—C7	179.3 (2)
C11—O4—C10—C8	171.6 (2)	C8—C7—C6—C1	−0.6 (4)
O3—C10—C8—C7	151.7 (3)	C8—C7—C6—C5	−179.9 (2)
O4—C10—C8—C7	−27.9 (3)	C1—C6—C5—C4	0.0 (4)
O3—C10—C8—C9	−26.2 (4)	C7—C6—C5—C4	179.3 (2)
O4—C10—C8—C9	154.2 (2)	C6—C5—C4—C3	1.0 (4)
C1—O1—C9—O2	179.5 (2)	C2—C3—C4—C5	−0.6 (4)
C1—O1—C9—C8	−3.3 (3)	O5—C14—C15—C16	−178.4 (2)
C7—C8—C9—O2	178.4 (3)	C13—C14—C15—C16	0.5 (4)
C10—C8—C9—O2	−3.8 (4)	C14—C15—C16—C11	−0.8 (4)
C7—C8—C9—O1	1.6 (3)	C12—C11—C16—C15	0.6 (4)
C10—C8—C9—O1	179.4 (2)	O4—C11—C16—C15	174.4 (2)
C9—O1—C1—C2	−177.2 (2)		

### Hydrogen-bond geometry (Å, º)

Cg1 and Cg2 are the centroids of the C1/C6/C7/C8/C9/O1 and C1/C2/C3/C4/C5/C6 rings, respectively.

**Table:**

*D*—H···*A*	*D*—H	H···*A*	*D*···*A*	*D*—H···*A*
C17—H17*B*···O5^i^	0.96	2.50	3.228 (3)	132
C12—H12···O2^ii^	0.93	2.48	3.353 (3)	156
C15—H15···O2^iii^	0.93	2.50	3.207 (3)	133
C3—H3···O3^iv^	0.93	2.47	3.272 (4)	145
C5—H5···*Cg*1^v^	0.93	2.82	3.303 (3)	114
C17—H17*C*···*Cg*2^vi^	0.93	2.96	3.709 (4)	136

Symmetry codes: (i) *x*−1/2, −*y*+3/2, −*z*+1; (ii) *x*−1, *y*, *z*; (iii) −*x*+2, *y*+1/2, −*z*+1/2; (iv) −*x*+3/2, −*y*+1, *z*−1/2; (v) −*x*, *y*+1/2, −*z*+3/2; (vi) *x*+3/2, −*y*+1/2, −*z*−1.

## References

[bb1] Bruker (2013). *APEX2*, *SAINT* and *SADABS*. Bruker AXS Inc., Madison, Wisconsin, USA.

[bb2] Chimenti, F., Secci, D., Bolasco, A., Chimenti, P., Bizzarri, B., Granese, A., Carradori, S., Yáñez, M., Orallo, F. & Ortuso, F. (2009). *J. Med. Chem*. 52, 1935–1942. 10.1021/jm801496u 19267475

[bb3] Devarajegowda, H. C., Palakshamurthy, B. S., Harishkumar, H. N., Suchetan, P. A. & Sreenivasa, S. (2013). *Acta Cryst.* E**69**, o1355–o1356. 10.1107/S1600536813020679 PMC3793833 24109420

[bb4] Kawase, M., Varu, B., Shah, A., Motohashi, N., Tani, S., Saito, S., Debnath, S., Mahapatra, S., Dastidar, S. G. & Chakrabarty, A. N. (2001). *Arzneim. Forsch./Drug Res.* **51**, 67. 10.1055/s-0031-1300004 11215328

[bb5] Lacy, A. & O’Kennedy, R. (2004). *Curr. Pharm. Des* **10**, 3797–3811. 10.2174/1381612043382693 15579072

[bb6] Macrae, C. F., Bruno, I. J., Chisholm, J. A., Edgington, P. R., McCabe, P., Pidcock, E., Rodriguez-Monge, L., Taylor, R., van de Streek, J. & Wood, P. A. (2008). *J. Appl. Cryst.* **41**, 466–470.

[bb7] Sheldrick, G. M. (2008). *Acta Cryst.* A**64**, 112–122. 10.1107/S0108767307043930 18156677

[bb8] Sheldrick, G. M. (2015). *Acta Cryst.* C**71**, 3–8.

[bb9] Sreenivasa, S., Srinivasa, H. T., Palakshamurthy, B. S., Kumar, V. & Devarajegowda, H. C. (2013). *Acta Cryst.* E**69**, o266. 10.1107/S1600536813001591 PMC3569796 23424542

[bb10] Traven, V. F. (2004). *Molecules*. 9, 50–66. 10.3390/90300050 PMC6147435 18007411

