# Supplementary figures and images for: Crystal structure of 4-meth­oxy­phenyl 2-oxo-2H-chromene-3-carboxyl­ate
Source: Acta Crystallogr E Crystallogr Commun. 2015 May 7;71(Pt 6):o374–5. doi: 10.1107/S2056989015006970 (PMC4459373; doi:10.1107/S2056989015006970)

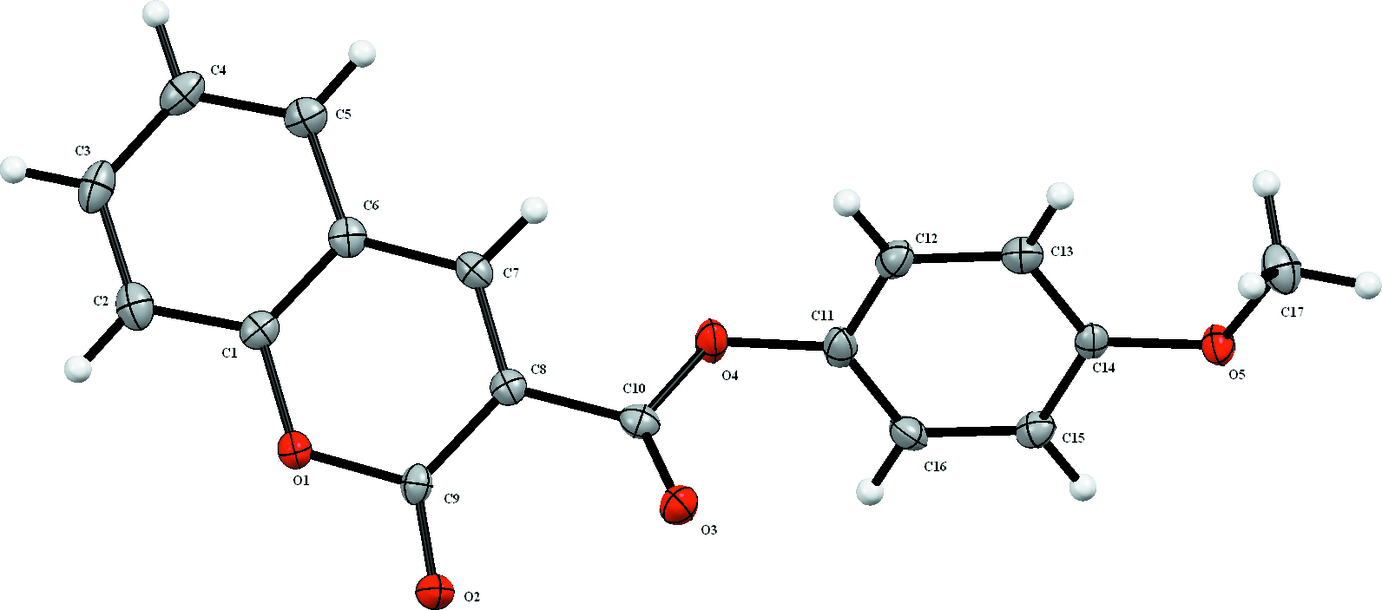

Supplement: Supplementary file 4 [file e-71-0o374-fig1.tif]

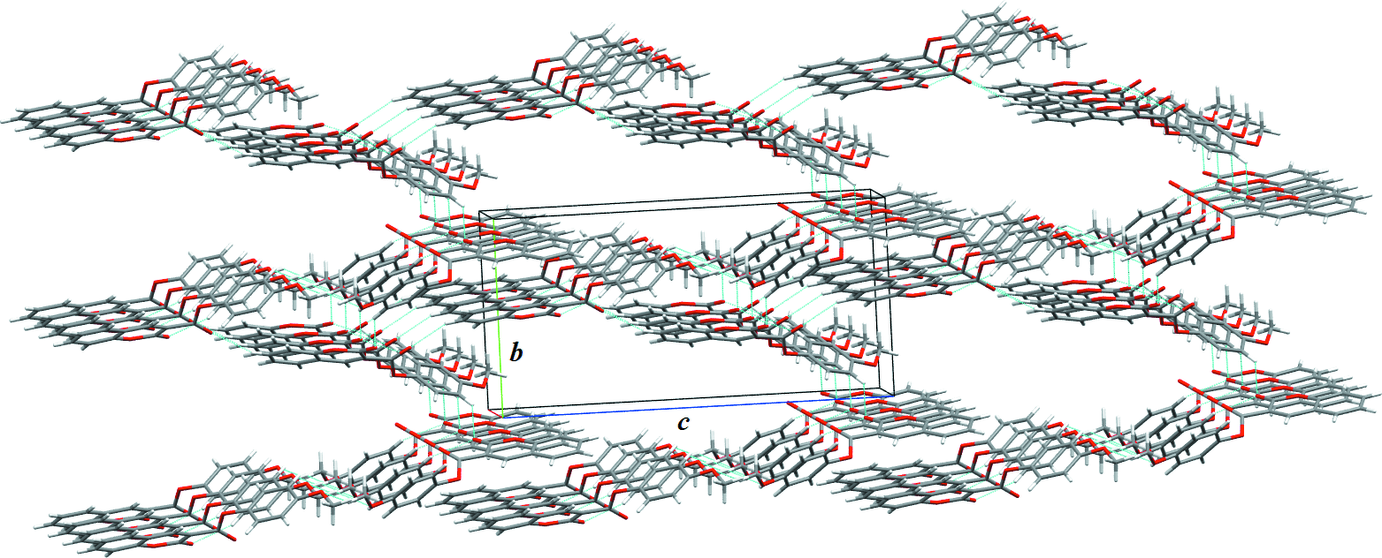

Supplement: Supplementary file 5 [file e-71-0o374-fig2.tif]

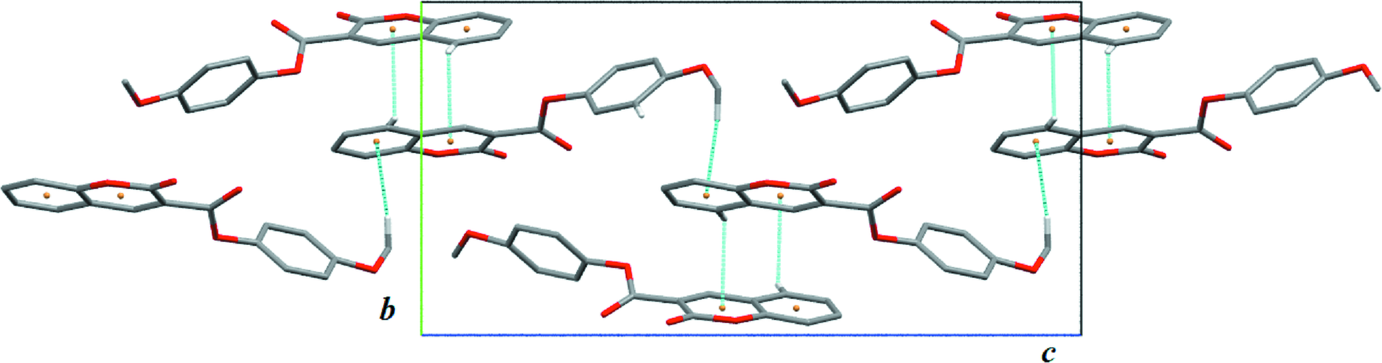

Supplement: Supplementary file 6 [file e-71-0o374-fig3.tif]
